# Lived experiences of CKD care in KZN: Barriers, facilitators, and practical realities

**DOI:** 10.4102/hsag.v30i0.2950

**Published:** 2025-09-09

**Authors:** Verosha Ramkelawan, Pretty Mbeje, Ntombifikile G. Mtshali

**Affiliations:** 1 School of Nursing and Public Health, College of Health Sciences, University of KwaZulu-Natal, Durban, South Africa

**Keywords:** chronic kidney disease, primary healthcare, guidelines adherence, implementation, healthcare professionals, chronic kidney disease management

## Abstract

**Background:**

Chronic kidney disease (CKD) is a significant public health concern in KwaZulu-Natal (KZN), exacerbated by a high burden of HIV, diabetes and hypertension, and compounded by socioeconomic barriers that limit access to preventative healthcare. With KZN accounting for 20% of South Africa’s dialysis patients, strengthening CKD management at the primary healthcare (PHC) level is crucial.

**Aim:**

The study used a qualitative phenomenological approach to explore healthcare providers’ (HCPs) lived experiences with CKD management in PHC settings in KZN, focusing on perceived barriers, facilitators and implementation realities.

**Method:**

A qualitative phenomenological approach was used to explore the lived experiences of HCPs managing CKD in PHC settings in KZN, South Africa. Through semi-structured interviews, the study examined perceived barriers, facilitators and real-world challenges influencing the implementation of CKD interventions and guideline adherence.

**Results:**

The study uncovered five key themes reflecting HCPs experiences with CKD guideline implementation at the PHC level: inconsistent guideline awareness and adherence, inadequate training, challenges in early detection because of limited resources, the importance of team-based care and patient involvement, and broader systemic and community-level barriers. Participants underscored the need for improved training, resource allocation and integrated care approaches.

**Conclusion:**

Implementation gaps stem from limited awareness, inadequate training and systemic barriers. Strengthening early detection, capacity building and team-based care is key to improving CKD management in PHC.

**Contribution:**

The study offers practical insights into implementation challenges, guiding policymakers and PHC managers in enhancing CKD care in resource-limited settings.

## Introduction

Chronic kidney disease (CKD) is a leading global cause of death, affecting over 840 million people, particularly in low- and middle-income countries (LMICs) (George & Kengne 2024; Okpechi et al. 2021). In South Africa (SA), despite improvements in healthcare through the primary healthcare (PHC) approach since 1994, the dual burden of non-communicable and communicable diseases continues to challenge the system (Mkhwanazi et al. 2023; Modjadji 2021; Wong et al. 2021). Chronic kidney disease disproportionately impacts women, older adults, and low- and middle-income earners, highlighting the urgent need for improved prevention and management strategies (George & Kengne 2024; Kovesdy 2022; Okpechi et al. 2021). The increasing prevalence of CKD is driven by ageing populations and risk factors such as obesity, hypertension and diabetes (Tangri et al. 2023). However, accurate CKD prevalence data in Africa are lacking, which hampers effective healthcare planning and policy development (George et al. 2021).

Chronic kidney disease often goes undiagnosed until stage V because of non-specific early symptoms, making early detection critical to preventing severe complications such as cardiovascular disease (Ayat Ali et al. 2021). While CKD awareness remains low, with less than 5% of individuals with early-stage CKD aware of their condition, early treatment, monitoring and referral can be guided by staging and novel risk assessments like albuminuria and glomerular filtration rate (GFR) (Chen, Knicely & Grams 2019). Effective interventions include annual screenings for high-risk individuals using blood pressure, glucose monitoring, urine dipstick tests and GFR estimation to identify early kidney dysfunction (Paget et al. 2015). The PHC practitioners play a crucial role in early CKD detection and management, which can delay or prevent the progression to end-stage kidney disease (ESKD) when coupled with lifestyle changes and appropriate treatments (Chironda & Bhengu 2019; Molaoa, Bisiwe & Ndlovu 2021). Chronic kidney disease is frequently underdiagnosed and undertreated because of low awareness, vague early symptoms and insufficient epidemiological data, making timely detection and policy responses challenging (Chen et al. 2019). The absence of systematic early detection measures at the PHC level exacerbates the issue, especially in the context of ageing populations and risk factors such as obesity, hypertension and diabetes (Ayat Ali et al. 2021; George & Kengne 2024). To mitigate the progression of ESKD and reduce the disease burden in high-risk populations, it is crucial to strengthen CKD monitoring and enhance PHC capabilities for early diagnosis, risk assessment and intervention (Chironda & Bhengu 2019; Paget et al. 2015). This study seeks to explore the lived experiences of healthcare providers (HCPs) managing CKD in PHC settings in KwaZulu-Natal (KZN). In addition, the study will identify the systemic, institutional and contextual barriers that hinder the successful implementation of these guidelines. The motivation for this research stems from the investigator’s observations of a persistent gap between policy and practice in PHC settings, evident in delayed CKD diagnoses and a growing number of patients presenting in advanced stages of the disease at tertiary facilities, often requiring urgent dialysis.

### Aim of the study

The aim of this study is to use a qualitative phenomenological approach to capture the lived experiences of HCPs managing CKD in PHC settings in KZN, South Africa and explore barriers, facilitators and practical realities of implementing CKD interventions and guidelines, as perceived by these HCPs.

## Research methods and design

### Research approach and design

This study utilised a qualitative phenomenological approach to deeply explore the lived experiences of HCPs involved in managing CKD within PHC settings in KZN. Phenomenology was chosen to uncover the perspectives, challenges and insights of HCPs actively engaged in implementing CKD care. Guided by a constructivist inquiry paradigm, the study recognised that the uptake of CKD guidelines is influenced by HCPs individual experiences and their interactions within the clinical environment. Trustworthiness was ensured through credibility, dependability, confirmability and transferability measures.

### Research setting

Primary healthcare forms the backbone of the public health system, providing essential services such as health promotion, disease prevention, screening, chronic disease management and referrals. In the context of CKD, PHC plays a critical role in early detection, monitoring of at-risk individuals, managing comorbidities such as hypertension and diabetes, and facilitating timely referrals to specialised care.

### Population, sample and sampling

A purposive sampling approach was used to select participants with relevant experience and knowledge, ensuring the richness of the data. Participants comprised HCPs working at the selected PHC centres, including doctors (*n* = 3), nurses (*n* = 8) and social workers (*n* = 1). These individuals were purposively selected because of their direct involvement in the care of patients with CKD and their integral roles within the multidisciplinary care team. Inclusion criteria required that participants be HCPs based at the identified facilities and actively engaged in the clinical management of patients with CKD. The number of participants available for the study was limited by staff shortages. These HCPs were involved in the management of CKD for a minimum of 6 months. Exclusion criteria were HCPs who were not involved in the management of CKD at PHC level.

### Data collection

Data were collected through semi-structured interviews with HCPs at three PHC facilities in KZN, representing both urban and township settings (Table 1). This helped to capture a wide range of experiences and challenges related to CKD management across different resource levels. The interviews were designed to explore how CKD is managed in PHC, how national guidelines are being applied in practice, and what barriers or supports exist for effective care.

**TABLE 1:** Healthcare providers’ at different sites.

Job tittle	Institution A (IA) – Urban	Institution B (IB) – Township	Institution C (IC) – Urban
Doctor	-	2 medical officers (1-female 1-male)	1 medical officer (female)
Nurse	4 (females)	3 (females)	1 (female)
Social worker	-	-	1 (female)
Other: specify	-	-	-

The researchers developed an interview guide with two main parts: one to collect basic participant information (such as job title and workplace) and another to focus on CKD care. Questions were guided by a central focus: understanding how CKD guidelines and interventions are being implemented in real-world PHC settings. Probing questions were used to explore deeper issues such as resource limitations, teamwork, training and patient involvement affecting its implementation.

Interviews were conducted in private settings to ensure confidentiality and lasted over a 3-month period. Field notes were taken and all interviews were audio recorded, with data collection continuing until no new themes were emerging (thematic saturation). This process ensured a rich and authentic understanding of the real-life factors influencing CKD care in PHC settings.

### Data analysis

Thematic analysis was used to analyse the qualitative data, in line with the study’s aim to explore how CKD is managed in PHC settings and how HCPs experience the implementation of national CKD guidelines. The analysis focused on the lived experiences of HCPs, particularly how they interact with existing guidelines and navigate the barriers and supports within their work environment.

To ensure depth and accuracy, the researcher read each transcript multiple times to become fully immersed in the data. Coding began with a mind-mapping approach to identify key ideas, recurring challenges and meaningful expressions from participants. Codes were refined through an iterative process and grouped into categories that reflected both individual experiences and broader systemic patterns. These categories were then synthesised into overarching themes that captured the practical realities of CKD care, including issues of resource availability, teamwork, training and patient engagement. Verbatim quotes were included to highlight participants’ voices and provide authentic insight into their perspectives.

### Techniques to enhance trustworthiness in qualitative data

To ensure the trustworthiness of the data, the study adhered to the four established criteria for qualitative rigour: credibility, dependability, confirmability and transferability. Credibility was achieved through member checking and peer debriefing. Dependability was ensured by maintaining a detailed audit trail of all research steps. Confirmability was addressed through reflexive journaling and independent coding by multiple researchers. Transferability was supported by providing rich, contextual descriptions of the study setting and participants.

### Ethical considerations

Data collection began after receiving ethical approval from the University of KwaZulu-Natal Biomedical Research Ethics Committee (BREC/00004880/2022), with additional permission from the provincial health department, the municipality and the PHC facility manager. Informed consent was obtained from all participants after a clear explanation of the study. Participants were anonymised using codes (e.g. P1, P2), and no identifying information was shared. All data, including consent forms, recordings and transcripts, were securely stored and accessed only by the researcher and supervisor.

## Results

This study explored the lived experiences of HCPs regarding the adoption and implementation of CKD management guidelines in PHC settings in KZN, South Africa. Five overarching themes emerged from the study.

### Theme 1: Awareness and adherence to chronic kidney disease guidelines

Healthcare providers expressed mixed experiences regarding CKD guideline awareness and application. Some reported easy access to clinical protocols via digital platforms and felt confident using them within their PHC roles. Others, however, were uncertain about where to find the guidelines or lacked clarity on how to apply them effectively. Several participants highlighted the need for further education and training, while a few admitted having little to no knowledge of the guidelines. These differing perspectives reveal inconsistencies in guideline access, understanding and use, underscoring the need for improved dissemination, structured support and ongoing capacity-building to ensure uniform adherence to CKD management protocols across PHC settings. The following narratives illustrate participants’ viewpoints:

‘I am not sure, but normally we use the Essential Drug List [*EDL*], that is what is guide us. So the EDL they update it now and then managing or optimising HPT [*ACE-i*], DM [*optimizing metformin or switching to injectables*].’ (Institution C- P2, female, Doctor)

‘There are guidelines implemented for the management of associated diseases and management of symptomatic treatment for CKD support. These guidelines are implemented according to human resources at a PHC setting and the level of training of PHC nurses.’ (Institution C- P1, female, nurse)

‘[*Laughs*] like I said the current guidelines, I don’t know which ones are you referring to you understand. Even when I ask my senior there nothing written down. Its based on EM guidance, says that this drug in contra indicated for CKD you see.’ (Institution C- P2, female, Doctor)

### Theme 2: Training and capacity building

Participants expressed a clear need for enhanced training and capacity-building initiatives to improve CKD diagnosis and management in PHC settings. While some HCPs were familiar with CKD guidelines, others felt current protocols lacked relevance or detail for routine PHC use. In-service training was widely viewed as a valuable tool to strengthen clinical confidence and improve care quality. Respondents emphasised the importance of regular, structured education sessions, especially for rotating staff and those without formal PHC training. Suggestions included outreach programmes, updated CKD signs and management training, and visual aids such as posters to reinforce learning. Overall, the following narratives highlight the critical role of continuous professional development in standardising care and improving patient outcomes:

‘Training the staff and educating the staff, you will see better diabetes mellitus care and improvement in patiens condition, posters on renal care is needed.’ (Institution C- P3, female, social worker)

‘… From time to time we can do the in-service for our staff as we are rotating what about those who are not PHC trained and they are supposed to work in MOPD and they have to see the acute, you will be alone in the room and have to see the patient, so if it is updated it will help the patient and it will also update the nurse.’ (Institution B- P4, female, nurse)

‘Standardised education and awareness of patients signs and consequences and all PHC’s, there should be more outreach programmes to inform staff about management and signs and symptoms.’ (Institution A- P3 female, nurse)

### Theme 3: Early detection and resource availability

Participants consistently emphasised the value of regular screening and adherence to CKD management guidelines in PHC settings. Tools such as the EDL and Jaccold assessment (jaundice, anaemia, cyanosis, clubbing, oedema, lymphadenopathy) were frequently used to assess symptoms such as oedema and anaemia. These tools, along with routine tests (e.g. creatinine levels, urine tests), were seen as critical for early detection and intervention, especially among high-risk groups such as patients with diabetes, hypertension or HIV.

However, despite awareness of best practices, knowledge gaps and resource constraints hindered optimal implementation. Some staff lacked clarity on CKD assessment protocols, and shortages in staffing, equipment and specialised support (e.g. dietitians, social workers) were common. These limitations often impacted care quality and follow-up, reinforcing the need for updated, stage-specific guidelines and better resource allocation. These resource constraints were commonly cited as barriers to effective CKD care. The following excerpts reflect participants’ views:

‘We conduct urine testing using the Jaccold assessment tool e.g assessment for edema, we measure the patients weight, We give dietary education, we do bloods, creatinine levels every 6 months for diabetic and hypertensive patients yearly, for HIV patients we monitor them 3 monthly for if they on anti-retro viral treatment.’ (Institution A- P3, female, nurse)

‘Dyslipidemia, monitoring proteinuria, Baseline potassium/ U/E, Monitoring fluid overload [*administration of Furosemide oral/IV*], Pain control, stopping nephrotoxic agents [*e.g. NSAIDs, TDF …*]’ (Institution A- P4, female, nurse)

‘[*Y*]es, but due to lack of resources and access to basic investigations and equipment are currently some challenges faced at a PHC level makes it difficult to monitor these patients, a more optimized guideline according to staging will benefit these patients …’ (Institution B- P5, male, doctor)

‘If we can, as they we can, as the medical staff we supply the treatment we can encourage the patients for compliance …’ (Institution C- P1, female, nurse)

‘Yes, that is what we need, they don’t know what is current. Especially with new machines …’ (Institution B- P4, female, nurse)

### Theme 4: Multidisciplinary collaboration and patient engagement

Multidisciplinary collaboration and patient engagement were assessed by determining the roles of HCPs, patient education and adherence to CKD management plans. Participants expressed concerns about the lack of clear, standardised guidelines for CKD management in PHC settings, leading to ambiguity around roles and responsibilities, which could result in fragmented care and inefficiencies. Some participants stressed the need for written guidelines to clarify referral processes and improve communication among HCPs. Multidisciplinary collaboration was identified as crucial for effective CKD care, with input from various HCPs, including physicians, nurses, dieticians and social workers. When roles are unclear, care integration becomes disjointed, potentially leading to suboptimal patient outcomes.

In addition, patient education and engagement were key components of successful CKD management. Participants highlighted the importance of informing patients about their condition and encouraging adherence to treatment plans, lifestyle changes and regular follow-ups, as educated patients are more likely to follow their care plans and achieve better long-term outcomes. The following narratives explained participants’ views:

‘Implementation of guidelines according to level of resourced facility [*e.g. Nutritionist or dietician referral*], implemented in conjunction with awareness programs for patients to adhere to treatment. In service training with PHC nurses to refer patients earlier to doctors. Improve referral process, some include optimizing Pharmacological support, Nutritionist referral, social worker or counsellor support.’ (Institution C –P1, female, nurse)

‘In PHC setting what we do is we try and do annual bloods with patients with normal renal function but what we do is as soon as we detect there is an abnormality obviously we have a look if they are diabetic, hypertensive they age as well and we try and optimize their chronic illnesses as well as HIV and stuff like that as soon as we pick it up we try and get history if there is any use of concomitant drugs as well if any other family history of that sort and that. What we see of lately is that we have a lot of patients who are also using a lot of traditional medication as well as energy drinks and so, so ours is that we counsel them and we encourage increased fluid intake and stop you know of whatever stuff might be and stopping drugs might also you know may worsen renal function or change to drugs that you know are renal friendly ya.’ (Institution C- P2, female, Doctor)

### Theme 5: Systemic barriers and community-based interventions

Participants experienced key systemic challenges such as staff shortages, limited medical supplies and inconsistent application of CKD guidelines. These barriers often led to adaptive strategies, including group-based education sessions and prioritisation of available treatments. Concerns were also raised about poor patient compliance and the lack of clear, written protocols hindered effective care and timely referrals. Despite these constraints, HCPs emphasised the value of community outreach, family education and awareness programmes to strengthen CKD care and improve patient outcomes in resource-limited PHC settings. The following narrative explains participants’ views:

‘Current guidelines are implemented but poor adherence and compliance from patients makes efficacy unreliable … due to lack of resources and access to basic investigations and equipment. Currently some challenges faced at a PHC level makes it difficult to monitor these patients, a more optimized guideline according to staging will benefit these patients.’ (Institution B- P5, Male, doctor)

‘[*E*]rr I think the staff shortage is a big thing everywhere, so I think that’s a hindrance for our sector at the moment it’s that there aren’t bodies available, now that they do patient education in group sessions but these are not as effective, that is unfortunately what it resort to ya.’ (Institution A- P1, female, nurse)

‘Mmm Okay, I think they [*guidelines*] are being implemented they are. But erm like I said its easier if there’s something written down because at the moment everyone does what they feel. You see also and that’s not the correct approach. Cause you end up losing some patients in the system or late referrals. neh.’ (Institution C- P2, female, Doctor)

‘Outreach and awareness programs from rehabilitation units is needed and family should be taught how to cope with this disease, there should be more things instituted like renal units to give a talk on kidney disease to create awareness and conduct outreach programs …’ (Institution A- P1, female, doctor)

‘I think its just that its shallow [*CKD management*] in the PHC level its shallow unlike when you refer to the hospital …’ (Institution B- P4, female, nurse)

Collectively, these themes highlight critical gaps in guideline awareness, training and resources, as well as the importance of multidisciplinary collaboration. Addressing these issues is essential for improving the adoption and implementation of CKD management interventions at the PHC level in KZN.

## Discussion

This study clarifies the conflicting experiences of HCPs regarding actions related to managing patients with CKD.

### Awareness and adherence to chronic kidney disease guidelines

Chronic kidney disease poses a growing public health burden that necessitates early detection and adherence to evidence-based management strategies to prevent its progression and associated complications (Francis et al. 2024). However, inconsistencies in CKD guideline awareness and application among PHC professionals remain a significant challenge. Neale, Middleton and Lambert (2020) identified several barriers to adherence, including time constraints, hesitancy to diagnose CKD, and dissatisfaction with existing protocols. Stengel et al. (2021) further illustrated these gaps, noting that only a minority of patients in the United States receive appropriate dietary guidance or reach target blood pressure levels, highlighting a disconnect between guideline knowledge and clinical practice.

This study aligns with findings from Bello et al. (2019), showing variable familiarity with CKD guidelines among HCPs, often because of limited access to updated resources. Structured training improves HCPs practice adherence (Singh et al. 2023). Integrating clinical pathways into electronic health records (EHRs) enhances the delivery of standardised, evidence-based care (Bartlett et al. 2019). Together, these strategies help mitigate the impact of training deficits. A multifaceted strategy, including accessible guidelines, embedded protocols and robust provider training, is essential to improve adherence and patient outcomes (Luyckx et al. 2024; Neale et al. 2020).

### Training and capacity building

In-service training and skill development are crucial for enhancing the ability of PHC professionals to diagnose and manage CKD effectively. Structured educational interventions have been shown to significantly improve HCPs knowledge, confidence and clinical practices in CKD care. Almaqhawi (2024) identified considerable gaps in PHC physicians’ knowledge and confidence in CKD screening, diagnosis and management, emphasising the need for continuous education to improve care quality and outcomes. Similarly, Tai, Foo and Ignacio (2024) found that educational interventions led to marked improvements in nurses’ knowledge, attitudes and practices in CKD care, contributing to higher-quality patient management. In addition, Ayat Ali et al. (2021) demonstrated that a nurse-led self-management support programme enhanced patients’ self-care behaviours and health outcomes, highlighting the value of equipping nurses with relevant skills for patient education and support.

Together, these studies emphasise the importance of ongoing, context-specific training for PHC providers. Tailored educational programmes can address existing knowledge gaps, strengthen clinical practices and support more effective and patient-centred CKD care.

### Early detection and resource availability

Effective screening and early detection are critical components of managing CKD and improving patient outcomes. Targeted screening strategies, particularly for high-risk populations, have been shown to enhance CKD detection, with one study reporting a higher detection rate (14.8%) in risk-based screening compared to population-based methods (8.0%) (Okpechi et al. 2022). Early CKD identification through diagnostic procedures such as estimated glomerular filtration rate (eGFR) and albuminuria tests is essential. However, limited access to these tools in LMICs poses significant challenges (George et al. 2022). Studies also highlight the importance of minimal-resource screening tools in resource-limited settings to prioritise individuals needing comprehensive CKD evaluation (Sammut-Powell et al. 2022).

The availability of essential resources, including diagnostic tools, plays a crucial role in the success of CKD early detection and management programmes. High-income countries, such as the United Kingdom, have implemented public health programmes like the National Health Service (NHS) Health Check, which aims to detect CKD and other conditions early, although the uptake remains suboptimal (McCracken et al. 2024). In LMICs, efforts to increase access to diagnostic tools and screening resources are essential to closing the care gap in CKD. Studies highlight the need to prioritise resource-efficient screening strategies that enhance early detection and improve treatment outcomes (Wen et al. 2025).

### Multidisciplinary collaboration and patient engagement

Multidisciplinary collaboration plays a crucial role in the effective management of CKD, as it ensures a comprehensive approach to patient care. The roles of various HCPs, including nephrologists, nurses, dietitians, social workers and primary HCPs, are integral to CKD management. Each professional contributes their expertise to different aspects of care, such as early diagnosis, dietary management, lifestyle modifications and psychosocial support. Effective communication and clearly defined roles within multidisciplinary teams are essential for enhancing patient care and ensuring holistic management of CKD (KDIGO 2024). Team-based care has been shown to improve patient outcomes, leading to better adherence to treatment plans, reduced hospitalisations and enhanced quality of life for patients (Abe et al. 2025; Kushner et al. 2024).

In addition to multidisciplinary collaboration, patient education and engagement are essential components of successful CKD management. Educating patients about their condition, the importance of adherence to treatment plans and necessary lifestyle changes can significantly improve health outcomes. When patients are well-informed, they are more likely to adhere to prescribed management plans, including medication regimens, dietary changes and regular follow-ups. Studies have shown that active patient engagement in their care leads to better self-management, reduced complications and slower progression of CKD (Lin & Hwang 2020).

### Systemic barriers and community-based interventions

Barriers to effective CKD care are largely driven by systemic, socioeconomic and institutional factors that impede both prevention and treatment efforts. Limited access to healthcare services in low-resource settings restrict early detection and ongoing management. Socioeconomic challenges such as poverty, low educational attainment and lack of healthcare access further delay diagnosis and lead to suboptimal care. In many LMICs, these issues are compounded by shortages of HCPs, especially nephrologists, and limited diagnostic tools, all hindering effective CKD management (Ramakrishnan et al. 2022). Institutional barriers, including inadequate infrastructure and medication shortages, are critical in worsening CKD outcomes (Luyckx et al. 2024).

Community-based interventions offer a viable solution to mitigate these challenges. These initiatives, which emphasise health education, lifestyle changes and early screening, are especially valuable in resource-limited areas. Community health workers are key to increasing CKD awareness and promoting early detection. Evidence demonstrates the positive impact of such interventions in improving CKD outcomes in underserved regions (Sharma et al. 2025).

### Limitations

As the study is limited to PHCs in a single province, its conclusions cannot be applied to all HCPs who treat patients with CKD. The researcher could only recruit a small number of participants from the various disciplines at PHC centres because of a staffing deficit.

### Recommendations for strengthening chronic kidney disease management at the primary healthcare level

Based on the study’s findings, the following recommendations are made to enhance CKD management in PHC settings and inform policymakers, health administrators and political leaders:

#### Enhance training and continuing education

There is a clear gap in HCPs understanding and application of CKD management guidelines. Facility managers and department heads should invest in regular, targeted training for PHC providers. This training should cover the latest CKD guidelines, early detection strategies, pharmacological and non-pharmacological interventions (such as diet and lifestyle changes), and clear referral protocols for advanced care.

#### Standardise chronic kidney disease management protocols

The study identified inconsistencies in CKD management practices across PHC centres. Health authorities should develop and implement standardised protocols to ensure consistent care delivery. These should include appropriate use of medications (e.g. ACE inhibitors, Angiotensin II Receptor Blockers [ARBs], Sodium-Glucose Co-Transporter 2 [SGLT-2] inhibitors), blood pressure control and regular monitoring of kidney function.

#### Implement electronic health records

Adopting digital health solutions such as EHRs can help track patient data, CKD risk factors, laboratory results, medications and disease progression. Electronic health records support timely interventions, facilitate continuity of care, and minimise data loss or mismanagement, especially in settings with limited staffing.

#### Strengthen health systems and policy integration

Policymakers should prioritise CKD within national health agendas and integrate CKD management into broader non-communicable disease (NCD) strategies. Emphasis should be placed on prevention, early detection and effective governance at the PHC level and a timely referral process. This approach will empower PHC providers and ensure they have up-to-date guidance for managing CKD in both clinical and community contexts.

#### Allocate adequate financial and material resources

Sufficient funding, personnel, medical supplies and equipment are essential to integrate CKD screening, diagnosis and management into routine PHC services.

These recommendations, grounded in the lived experiences of PHC HCPs, aim to address current gaps and support the effective implementation of CKD management guidelines at the primary care level. Enhancing CKD management at the PHC level, through early detection, adherence to clinical guidelines, capacity building and increased patient engagement, can help reduce CKD prevalence, slow its progression and improve overall health outcomes.

## Conclusion

This study evaluated how CKD management interventions are currently implemented in PHC settings in KZN and assessed their alignment with national guidelines. The findings highlight significant gaps in guideline awareness, inconsistent adherence, limited training and resource constraints among HCPs. Systemic and contextual barriers further hinder the effective uptake and implementation of CKD guidelines. Addressing these challenges through enhanced provider training, standardised protocols, improved resource allocation and integrated, multidisciplinary care will be essential for strengthening CKD management at the PHC level. These targeted interventions can improve early detection, promote consistent care and ultimately reduce the burden of CKD in KZN.
